# Effect of Progressive Step Marching Exercise on Balance Ability in the Elderly: A Cluster Randomized Clinical Trial

**DOI:** 10.3390/ijerph18063146

**Published:** 2021-03-18

**Authors:** Pairaya Sitthiracha, Wichai Eungpinichpong, Uraiwan Chatchawan

**Affiliations:** 1 School of Physical Therapy, Faculty of Associated Medical Sciences, Khon Kaen University, Khon Kaen 40002, Thailand; p.superboboro@gmail.com (P.S.); pomuraiwan67@gmail.com (U.C.); 2 Research Center in Back, Neck, Other Joint Pain and Human Performance (BNOJPH), Faculty of Associated Medical Sciences, Khon Kaen University, Khon Kaen 40002, Thailand

**Keywords:** balance exercise, elderly, exercise program, falls prevention exercise

## Abstract

Exercise may reduce the risk of falls in the elderly. The objective of the study was to determine the effect of a progressive step marching exercise (PSME) program on balance ability, lower limb muscle strength, aerobic capacity, quality of life, and fear of falling in the elderly. A cluster randomized controlled trial was selected where 30 elderly participants (aged 69 ± 3 years) from a community were supervised while performing a PSME program and 30 (aged 70 ± 3 years) from another community were assigned to a control group. All participants in both groups underwent timed up and go test, one leg standing test, five time sit to stand test, two minutes step test, World Health Organization Quality of Life-Thai version, and fall efficacy scale Thai version at baseline, after exercise at 4 and 8 weeks. The PSME group underwent the program for 8 weeks while those in the control group were instructed to continue their normal activity. After training, the PSME showed significant improvement (*p* < 0.05) in all parameters except one leg standing and two minutes step test when compared to the control group (*p* < 0.05). In conclusion, the PSME program could improve balance ability, lower limb muscle strength, quality of life, and fear of falling in the elderly.

## 1. Introduction

Nowadays, the number of older persons in the world is increasing [1]. In Thailand, the number of elderly aged 60 years old and over has grown significantly and will continue to do so in future decades. Aging is related to functional decline in sensory and motor processes that can cause balance deficits in the elderly [2,3]. These systems can induce physiological problems like falls. Falls are the third leading cause of death in the elderly population [4]. In Thailand, about one thousand death are caused by falls. Furthermore, falls are related to severe problems in the elderly such as hip fractures, fear of falling, disability, and mortality [5,6,7,8,9].

According to the World Health Organization (WHO) recommendation, the elderly aged over 65 years should perform moderate intensity physical activity for at least 150 min per week [10,11]. Besides, performance of fall prevention exercises by the elderly population is also recommended by WHO [1]. Numerous studies have found that dance-based exercise positively affects physiological changes that may affect intrinsic risk factors of falls in the elderly [12].

In this study, progressive step marching exercise (PSME) is a new balance training program which mainly designed to enhance a balance ability, lower limb muscle strength, aerobic capacity, quality of life, and fear of falling by adhering to balance training method recommendations [13,14]. The program is mainly designed to improve exercise adherence and decrease exercise boredom by including music which may apply for those who have limited space for exercise [15,16].

The PSME program could be affected by some physiological and psychological aspects in the elderly. An increasing ground reaction force around 1 to 1.2 percent of body weight may improve sensory and motor mechanisms resulting in balance improvement [17]. Because of PSME’s movement patterns, repetitive movement of knee joint and lower muscles may affect other systems by improving muscle strength and aerobic capacity. In the psychological aspect, group exercise can affect the quality of life level in most aspects such as physical, psychological, and social aspects [18,19,20]. Fear of falling in the elderly can be decreased by exercise intervention and balance improvement [21,22]. The purpose of the study was to determine the effect of progressive step marching program on balance ability, lower limb muscle strength, aerobic capacity, quality of life, and fear of falling in the community-dwelling elderly. The progressive step marching exercise in the study was modified to manage intrinsic risk factors of falls in the elderly such as balance deficit, muscle weakness, and mobility limitation. Furthermore, the program may improve psychological aspects such as quality of life and fear of falling in the elderly.

## 2. Materials and Methods

### 2.1. Study Design and Participants

The study was a cluster randomization design in twelve villages. Sai-moon subdistrict was selected as a setting community-dwelling with similar participant demographic data such as chronic disease, socioeconomic, exercise, and education level. Twelve villages in the Saimoon sub-district (Amphoe Nam Phong, Khon Kaen, Thailand) were selected for the study. An investigator prepared twelve village numbers in concealed envelopes then two envelopes were selected. The two selected communities were assigned to an exercise group (PSME) and a control group by the investigator.

**Figure 1:**
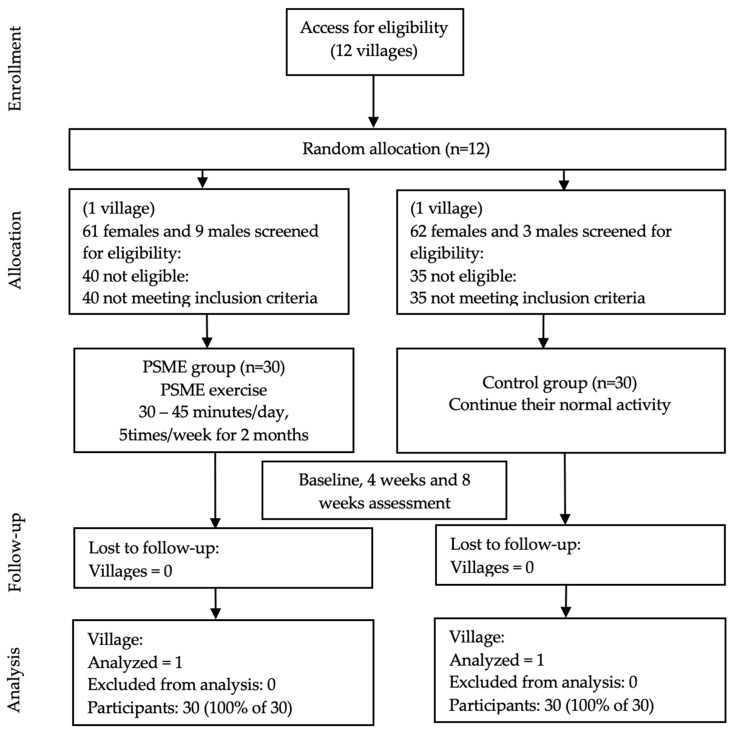
CONSORT flow diagram displaying cluster randomized clinical trial participant pathways through the 8 weeks study. PSME = Progressive step marching exercise.

Participants in the control group were instructed to continue their regular activity for 8 weeks. They did not receive any PSME training. Details of the subject recruitment and allocation process of the study are summarized in Figure 1. The study received approval from the Center for Ethics in Human Research, Khon Kaen University (HE622102). The participants gave informed consent before participating in the study.

### 2.2. Progressive Step Marching Exercise

The PSME was designed to emphasize the effect of marching and adhere to balance training principles [14,23]. The program’s advantage is a marching movement that moves knee joints about 45 degrees while participants listen to Thai music played at different speeds. The music was included to increase exercise adherence and decrease exercise boredom [15,16]. The PSME primary movement pattern is step marching and other movement patterns with a ratio of 80 and 20 percent, respectively. The study’s other movement patterns include walking (forward and backward directions) and step touch. Thai music with between 70 to 110 beats per minute (bpm) was selected for the study. The program was set to gradually increase music speed to three levels (Table 1).

### 2.3. Intervention

All participants in the PSME group participated in an exercise program for 8 weeks. The program was conducted five times per week for 35–45 min per session. The exercise program consisted of stretching, PSME program, and cool-down. The warm-up and cool-down consisted of general stretching on both sides for 5 s for each pose in the upper (neck, shoulder, trunk, elbow and wrist joints) and lower extremity (hip, knee, and ankle) muscles.

During the first month, the exercise duration was 30 min with a brief 5 min rest after the first 15 min of the program. The exercise movement patterns are marching in place, marching backward, marching forward, marching to left and right which must contain at 80 percent of all exercise movement patterns (Appendix A). Exercise duration was set as 35 min and increased to 45 min in the second month with 5 min rest between trails. Participants in the control group were instructed to continue their regular activity for 8 weeks. 

### 2.4. Outcomes Measurement

The psychological and physiological outcomes of the participants were assessed at the beginning, 4 weeks, and 8 weeks of the exercise program by outcome assessors blinded to the group each participant belonged to. According to physiological outcomes, participants received an explanation of the test and performed the test for one trial for familiarization.

#### 2.4.1. Cognitive Impairment

Before recruiting participants, they received cognitive impairment screening by using the Mini-Mental State Examination (MMSE). The elderly who had MMSE scores below the average cognitive level were excluded from the study. The Thai version questionnaire was developed from an original version to measure cognitive impairment in Thai people [24,25]. The interpretation is dependent upon the patient’s level of education. The total score possible is 30; the cut-off score for elderly who completed elementary schooling is 22 and, for those who did not, it is 17. The cut-off score for illiterate participants is 14, and for them, a total score of 23 is used instead. After cognitive impairment screening, all participants were assessed by using the following tests.

#### 2.4.2. Quality of Life

The WHOQOL–BREF–THAI has 26 questions divided into four domains: physical health, psychological, social relationships and environmental. The environmental domain consists of facets such as financial resources, health and social care, home environment, transportation, and physical environment. The four domain questions are seven questions screening physical health, six questions screening mental health, three questions screening social relationships, and eight questions screening environmental health. The answers are divided into five levels: none, little, middle, much, and very much. The scores on the physical health assessment were divided into three levels: 7–16 points, low level; 17–26 points, middle level; and 27–35 points, high level quality of life. The physical health assessment scores were divided into three levels: 7–16 points, low level; 17–26 points, middle level; and 27–35 points, high level quality of life. The mental health assessment scores were divided into three levels: 6–14 points, low level; 15–22 points, middle level; and 23–30 points, high level mental quality of life. The social relationship assessment scores were divided into three levels: 3–7 points, low level; 8–11 points, middle level; and 12–15 points, high level of quality of social relationships. The environmental factors scores were divided into three levels: 8–18 points, low level; 19–29 points, middle level; and 30–40 points, high level environmental factors. The overall interpretation of life quality was divided into three levels: 26–60 points, low level; 61–95 points, middle level; and 96–130 points, high level quality of life [26]. 

#### 2.4.3. Fear of Falling

The Fear of Falling (FES-I) in Thai language test developed by Thiamwong and colleagues was used in the study to assess participants’ fear of falling [27]. The questionnaire includes 16 daily indoor, outdoor, and social activities. The item scores range from 1 (not at all concerned about falling) to 4 (very concerned). Participants are asked to complete the questionnaire. The total score range is 16 to 64. Score 16–19 means low levels of concern, score 20–27 means a moderate level of concern, score 28–64 means a high level of concern [28]. 

#### 2.4.4. Timed Up and Go Test

The Timed Up and Go Test (TUGT) was modified from the Get-Up and Go Test [29]. The timed up and go, the time that patients required to rise from an armchair and walk 3 m, turn, walk back and sit again was observed [30]. Bischoff and colleagues suggested that community-dwelling older adults, age between 65 and 85 years, should be able to perform the TUGT in no more than 12 s [31]. Chantanachai and colleagues found that Thai elderly who have TUGT scores less than 10.5 s can be divided into fallers and non-fallers (74% sensitivity and 57.7% specificity) [32].

#### 2.4.5. One Leg Standing Test

The One Leg Standing Test (OLST) was used to assess an intervention such as balance exercise and assess fall risk in the elderly [33]. Participants were tested under opened eyes and closed eyes conditions for a maximum time of 45 s. They were asked to stand barefoot on their limb of choice, with a raised foot not touching the ankle of the stance leg. The participant was instructed to cross their arms over the chest. The investigator used a stopwatch to measure the amount of time the subject was able to stand on one limb. Time commenced when the subject raised the foot off the floor. Time ended when the subjects either: (1) used their arms (i.e., uncrossed arms), (2) used the raised foot (moved it toward or away from the standing limb or touched the floor), (3) moved the weight-bearing foot to maintain balance (i.e., rotated the foot on the ground), (4) a maximum of 45 s had elapsed, or (5) opened their eyes during closed eyes trials. The subjects performed three trials with opened eyes and three trials with closed eyes, alternating between the conditions [34]. 

#### 2.4.6. Five Times Sit to Stand Test

The Five Times Sit to Stand test (FTSST) was used to evaluate the times that participants take to go from a sitting position to standing five times. FTSST is related to greater deficits in instrumental activities of daily living and balance disorders in older adults [35,36]. This test is used to evaluate lower extremity muscle strength while sitting to standing [37,38,39]. FTSST requires a stopwatch and a 43 cm height standard armless chair. The prescription of FTSST are as follows; participants sit on the chair with their back upright at 90 degrees against the chair and their feet placed flat on the floor at 10 cm behind the knees. Participants were instructed to stand up with the hip and knees in full extension and sit down five times at the fastest and safest speed they can. An assessor will start recording the time at the word “Go” until the participant’s back touches the chair’s backrest on the fifth repetition [37]. Subject performed three trials/test and the average performance over the three trials was used for data analysis.

#### 2.4.7. Two Minutes Step Test

The Two Minutes Step Test (2MST) was used to assess the aerobic capacity by alternate aerobic endurance tests for use when space limitations or weather prohibits taking the 6-minute walk test. In the 2-minute step test, each participant raises each knee to a point midway between the patella and iliac crest. The score was counted by the number of full steps completed in 2 minutes. The score is the number of times the right knee reaches the required height [40]. 

#### 2.4.8. Statistical Analysis

Sample size and percent drop-out rate were calculated from a previous study that used dance-based exercise in the elderly [41]. They determined that the average (standard deviation) pre-test and post-test timed up and go test times were 12.7 (5.9) and 11.7 (14.5) seconds in the dance-based and control groups, respectively [42,43]. Thus, the study should have participants at least 36 persons in the exercise and control groups.

Descriptive statistics was used to describe baseline demographics and findings of the study. A Kolmogorov-Smirnov calculation was used to ensure the normal distribution of the data. The independent t-test was used to determine the differences of balance ability, lower limb muscle strength and aerobic capacity at pre-test and post-test between control and exercise group. Two-way repeated ANOVA was used to determine the differences of all parameters in pre-test, 4- and 8-week assessment in each group. Analysis of covariance (ANCOVA) analysis of covariance was used to assess differences between two groups at 4 and 8 weeks.

Differences were considered at the *p* < 0.05 level. Cohen’s d statistic was used to compute an effect size. The effect size less than 0.2 was considered to reflect a negligible difference, at least 0.2 to at most 0.5 a small difference, at least 0.5 to at most 0.8 a moderate difference, and at least 0.8 a large difference [44]. All calculations were performed with SPSS version 23.0 (SPSS Inc., Chicago, IL, USA). 

## 3. Results

Participant’s demographic data and health status in mean and standard deviation are shown in Table 2. There were sixty participants in the PSME and control group, with average ages (mean ± SD) of 69 ± 3 and 70 ± 3 years, respectively. In the PSME group, the number of females and males were 26 and four participants, with average weight, height, and body mass index (54.9 ± 8.9 kg., 154.27 ± 6.38 cm, and 23.0 ± 3.7 kg/m^2^), respectively. In the control group, the number of females and males was 27 and three participants with average weight, height, and body mass index of 53.1 ± 8.9 kg, 153.17 ± 6.41 cm, and 22.6 ± 3.6 kg/m^2^, respectively. Participant’s demographic data thus did not differ between the two groups.

Most participants were women, with married status, primary education level, and who had not done any aerobic exercise in the past 6 months. In terms of other variables, most factors were found to be equally balanced between the two groups. Three participants in the exercise group were lost to follow, so a single imputation method was used to take into account in all outcome data.

### 3.1. Timed Up and Go Test

A within-group comparison revealed a significant decrease in time scores testing at 4 (10.6 ± 1.4; *p* < 0.001) and 8 weeks (10.4 ± 1.5; *p* < 0.001) when compared to baseline data (11.9 ± 1.6) as shown in Table 3. In the control group, no significant improvement was found at 4 (11.4 ± 1.0) and 8 weeks (11.1 ± 1.0) when compared to the baseline data (11.6 ± 0.8) (Table 2). In between-group comparison, the PSME group showed an improvement at 4 (Adj. mean = 10.5, MD = −1.0, *p* < 0.001) and 8 weeks (Adj. mean = 10.3, MD = −0.9, *d* = 0.5; *p* < 0.001) when compared to the control group (Table 4).

### 3.2. One Leg Standing Test

One leg standing was performed under two conditions, that is with opened eyes and closed eyes. In within-group comparison, only the time score in the PSME group increased (Table 3). Nevertheless, the time score in closed eyes condition in both groups was not significantly improved after 8 weeks of exercise (Table 4).

### 3.3. Five Times Sit to Stand Test

Participants in the PSME group had improved lower limb muscle strength from baseline (13.3 ± 3.0) when compared to 4 weeks (9.4 ± 1.5; *p* < 0.001) and 8 weeks (8.2 ± 1.0; *p* < 0.001). Nevertheless, in the control group a significant decrease in the lower limb muscles strength from baseline (9.2 ± 1.9) to 8 weeks (10.7 ± 2.8; *p* < 0.001) (Table 3) was also found. In a between-group comparison by using ANCOVA, the PSME group exhibited a great lower limb muscle strength from baseline (Adj. mean = 10.7) until 4 weeks (Adj. mean = 8.4, MD = −1.2; *p* < 0.001). Besides, the muscle strength continuously improved to 8 weeks with a large effect size (Adj. mean = 7.8, MD = −1.4, *d* = 1.5; *p* < 0.001) (Table 4).

### 3.4. Two Minutes Step Test

Aerobic capacity in the PSME group exhibited an improvement at 4 and 8 weeks. The two minutes step test (2MST) revealed a significant improvement from baseline (mean ± SD, 62.5 ± 12.3) to 4 (77.7 ± 11.0; *p* < 0.001) and 8 weeks (84.3 ± 9.6; *p* < 0.001) (Table 3). The development in aerobic capacity also found in the between-group comparison was 68.8 to 87.0 steps. Nevertheless, this difference was not significant when compared to the control group (Table 4).

### 3.5. Quality of Life

The WHOQOL–BREF–THAI questionnaire was selected as a quality of life outcome measure in the study. In the within-group comparison, the results showed that participants in the PSME group had significantly improved their quality of life from medium to high after completing the PSME program. The quality of life domains are included physical domain (25.1 ± 3.9 to 30.8 ± 2.7; *p* < 0.001), psychological domain (20.8 ± 4.8 to 26.2 ± 2.8; *p* < 0.001), and overall domain (92.8 ± 13.6 to 110.4 ± 11.4; *p* < 0.001) (Table 5). The social relationship and environmental domains are also significantly improved after doing the program, but the level of quality of life did not change simultaneously.

A difference between the two groups was found in all quality of life domains (*p* < 0.05). After doing the exercise, participants seem to increase their physical domain, psychological domain, and overall quality of life from medium to good, with significant differences between the two groups. The other domains included social relationships and environmental domains and are also significantly different in the quality of life level compared to baseline data. The large effect size was found in the physical domain (*d* = 1.3) and overall domain (*d* = 1.0), whereas other variables have a medium to large effect size (Table 6).

### 3.6. Fear of Falling

The fear of falling level seems to decrease after doing the PSME program for 8 weeks. The participants in the PSME group displayed significantly decreased levels of concern (28.2 ± 5.9 to 21.3 ± 4.3 scores, *p* < 0.001) (Table 5). Furthermore, at 8 weeks of exercise, participants in the exercise group had significantly decreased fear of falling levels from baseline (Adj. mean = 26.4) to 8 weeks with a large effect size of the PSME program (Adj. mean = 21.0, MD = −5.73, *d* = 0.93; *p* < 0.001) when compared to the control group (Table 6). Nevertheless, the fear of falling level does not change from baseline until 8 weeks of exercise.

## 4. Discussion

In the present study, there was no comparison of PSME with other exercise modalities. The objective of the study was to determine the effect of the PSME program on physiological and psychological aspects in the elderly who have a risk of falls. The PSME program seems to affect balance ability by decreasing time in the TUGT, lower limb muscle strength by decreasing time using the FTSST. The quality of life and fear of falling also showed an improvement after finishing the program. However, the PSME group participants did not improve in the time using one leg standing test (OLST). In the control group, participants did not show improvement in any of variables except the FTSST.

### 4.1. Balance Ability in the Elderly

The PSME program seems to improve the participant’s balance ability, which was evident in the decreasing time score of TUGT at 4 and 8 weeks. It may be indicated that the PSME program is enhanced balance performance and functional ability when compared with the control group (Table 4). The magnitude of improvement found at 4 and 8 weeks, which is considered minimally clinically important (MCID), was between 0.8 to 1.9 s [45,46].

The findings of the study are similar to those of several studies that have investigated 8 weeks of dance-based exercise. The several types of dance-based exercises such as ballroom dance and Spanish dance showed an effective result on balance ability by time decreasing in TUGT (*p* < 0.05) with a large effect size (*d* = 1.2) [47,48]. Furthermore, the study results are similar to those of other studies that revealed the effect of dance-based exercise on balance ability, which had a different intervention period and frequency ranging from 6 weeks to 12 months [49]. These interventions included ballroom dancing, low-impact exercise, and Tai-chi [49,50,51].

According to the effect of PSME on the balance system, it may enhance both mechanoreceptor and proprioceptor strength in the feet by increasing the ground reaction force (1–1.3% of body weight) [52,53]. These receptors are played a role in the postural control system which may correct the posture while doing a marching movement [54]. Furthermore, the PSME program may improve muscle strength when using repetitive movements to overcome the bodyweight resistance and result in the motor unit recruitment and increased firing rate in the muscles [55,56]. Therefore, this effect would increase neural feedback from the cutaneous receptors to the central nervous system and possibly contribute to improved postural control [57,58].

In the OLST variable, opened eyes and closed eyes condition results tended to decrease after 8 weeks of exercise. Nevertheless, no significant changes were observed for those variables. The static balance can be defined as the ability to maintain stability on a fixed, firm, and unmoving base of support [59]. In the study, no improvement was found for the OLST which may be attributed to various factors. Firstly, the high standard deviation at baseline may indicate the wide range of the data. When doing the OLST variable, the participant needs to stand on one leg with a limit time of 45 s. Secondly, the difference between movement patterns between the PSME and other dance-based exercises such as flamenco dance style. The flamenco dancing includes simple flamenco dance steps (forward, backward, transversal, and rotational), sevillanas, and ballet steps which can improve static balance in opened eyes condition [48]. Besides, Shigematsu and colleagues also revealed the effect of 12 weeks (60 min/3 times/week) dance-based exercise (single leg, squatting, marching, heel touching, targeted balance, strength, locomotion, agility, and motor processing) in the elderly. They found that participants in exercise groups improved their static balance when tested by OLST in closed eyes conditions [60]. Those movement patterns may alter the static balance ability more than the PSME program.

### 4.2. Lower Limb Muscle Strength

These research findings showed that participants who underwent the PSME program had significantly improved lower limb muscle strength at 4 and 8 weeks compared to the control group. The FTSST is associated with lower limb muscle strength and functional ability in the elderly with a large effect (Cohen’s *d* = 1.55) [61]. A previous study showed a relationship between repetitive single leg standing and muscle activation. The relationship could enhance lower limb muscle activation and torque production in the muscles [50,51]. Furthermore, dance-based exercise might improve muscle morphology such as fascicle length and muscle thickness [47]. Several studies also showed that bodyweight exercise and muscle strength might improve the lower limb muscle strength in the elderly by increasing the motor unit recruitment and motor firing units in the muscle fiber [56,62,63].

According to the fall risk factor in the elderly, muscle weakness is the most impactful risk factor (OR-RR 4.4 range 1.5–10.3) [64]. These research findings implied that PSME can improve lower limb muscle strength which may increase functional abilities and may reduce the rate of falls in the elderly. Nevertheless, the changes score in the study (1.38 s at 8 weeks) was not associated with the MCID value that was evaluated on the elderly who have a vestibular disorder (more than 2.3 s) [65]. These findings may be explained by the different participant’s characteristics. In the study, the participant did not have any vestibular balance disorder according to the exercise precautions. The PSME program, which performed in repetitive marching movement for 8 weeks may lead to neural adaptation and muscle hypertrophy in the lower limb muscles. Besides, the exercise that has a repetitive movement with or without additional weight may improve muscle strength and intermuscular coordination [55].

### 4.3. Aerobic Capacity

After practicing the PSME program for 8 weeks, the exercise group improved their aerobic capacity, but no significant difference was found in this group. The result of the study does not relate to other dance-based exercise studies. Holmerová and colleagues established 12 weeks of ballroom dance exercise may enhance cardiovascular endurance in the elderly [66]. Furthermore, other dance-based exercises at 8 and 10 weeks also improved exercise endurance that was measured by a 6-minute walk test [47,67].

This lack of improvement in the exercise group may be due to test sensitivity, exercise duration, and data normality. Firstly, the 2MST was developed to measure aerobic capacity in the elderly who have a physical impairment which different from the population of this study. Secondly, at 8 weeks of exercise, the PSME group seems to improve their aerobic capacity when compared to baseline data (Table 3). Thirdly, the wide range of the standard deviation at 8 weeks of exercise in the PSME group may affect the statistical analysis result compared to the control group. One may conclude that the PSME exercise can alter the cardiovascular system in the participants who underwent the program.

### 4.4. Quality of Life

The results of the study established that the PSME program seems to improve the quality of life in all domains and decrease the fear of falling level when compared to the control group. The participants in the PSME group significantly improved their quality of life in overall domains including their physical (25.1 to 30.8), psychological (20.8 to 26.2), social relationships (11.2 to 11.9), environmental (24.6 to 24.4), and overall domains (92.8 to 110.4). These research findings are similar to those of other studies. Previous studies showed that dance-based exercises such as boxing dance and resistance exercise could be enhanced the quality of life level in some domains after 12 weeks of exercise [19,20].

### 4.5. Fear of Falling

The level of concern in the fear of falling variable was decreased when compared to the control group. In the study, their fear of falling level of participants in the PSME group seemed to decrease from high to medium level when compared to the control group. However, participants in the control group were in the medium level of concern until 8 weeks of the intervention. The fear of falling level can limit the physical and psychological problems in the elderly [68,69]. Besides, a systematic review study showed that exercise interventions in the elderly who lived in community-dwelling possibly reduce the fear of falling [21]. The dance-based exercise that used bodyweight as a resistance factor reduced the risk of falls in elderly aged 65 years old or above [22].

The first limitation of the study is physical activity control in both groups that may affect to physical performance of the participants. Any future study should be conducted with an accelerometer for monitoring the participant’s activity [70]. According to group exercise in the PSME group, a difference in social interactions between the groups may affect the quality of life level. In any further study, participants in both groups should be assigned to similar social interactions. Besides, cluster randomization was selected to allocate the participants in the study that may contribute to bias sampling and sampling errors. However, the allocation method also has a good advantage in that it prevents contamination between the two groups.

The current study is the first study that has conducted the PSME program in the elderly to improve balance ability, lower limb muscle strength, quality of life, and fear of falling. Besides, the PSME exercise that provides a repetitive ground reaction force to the foot through the bones may induce the adaptation to a bone mass in the elderly. Therefore, future research should investigate the physiological, biomechanics, and body composition aspects of the PSME program such as muscle activation, ground reaction force at different speeds, and bone densitometry.

## 5. Conclusions

The purpose of the study was to determine the effect of the PSME program on the community-dwelling elderly aged between 65 to 75 years old. After undergoing the program for 8 weeks (5 times/35–45 min/weeks), the elderly had significantly improved balance ability, lower limb muscle strength, quality of life, and fear of falling. Therefore, the PSME program could be conducted as an exercise for the elderly who live in community-dwelling scenarios to reduce the risk of falls, improve quality of life, and reduce fear of falling.

## Figures and Tables

**Table 1:** Progressive marching exercise program level.

Time (Week)	Level	Beats per Min			
		70–80	80–90	90–100	100–110
1–2	Level 1	10 min.	10 min.	-	-
3–5	Level 2	-	15 min.	15 min.	-
6–8	Level 3	-	-	15 min.	15 min.

**Table 2:** Participant’s demographic data and health status.

Characteristics	PSME Group (*n* = 30)	Control Group (*n* = 30)	*p*-Value
**Age (years)**	69 ± 3	70 ± 3	0.258 ^a^
**Weight (kg)**	54.9 ± 8.9	53.1 ± 8.9	0.438 ^a^
**Height (cm)**	154.27 ± 6.38	153.17 ± 6.41	0.396 ^a^
**Body mass index (kg/m^2^)**	23.0 ± 3.7	22.6 ± 3.6	0.692 ^a^
**Gender**			
**Female**	26 (85.8%)	27 (89.1%)	0.688 ^b^
**Male**	4 (14.2%)	3 (10.9%)	
**Marital status**			
**Single**	1 (3%)	0	0.382 ^b^
**Marry**	19 (63.3%)	16 (53.3%)	
**Widow/Divorced/Separated**	10 (33.3%)	14 (46.7%)	
**Education level**			
**Primary education**	29 (97.6%)	28 (93.2%)	0.554 ^b^
**Secondary education**	1 (3.3%)	2 (6.6%)	
**Underlying disease**			
**No underlying disease**	18 (60%)	22 (73.3%)	0.249 ^b^
**Diabetes mellitus**	8 (26.6%)	3 (10%)	
**Hypertension**	4 (13.32%)	5 (16.7%)	
**Others**	0	0	
**Exercise**			
**Never**	24 (80.02%)	19 (63.3%)	0.152 ^b^
**0–60 min/week**	6 (19.98%)	11 (36.7%)	
**Smoking**			
**Never**	28 (93.2%)	29 (96.7%)	0.554 ^b^
**Rarely and Regularly**	2 (6.8%)	1 (3.33%)	
**Drinking**			
**Never**	28 (93.3%)	28 (93.3%)	1.000 ^b^
**Rarely and Regularly**	2 (6.7%)	2 (6.7%)	
**Falls history (past 6 months)**			
**No falls**	27 (89.91%)	28 (93.3%)	0.64 ^b^
**Falls**	3 (10.09%)	2 (6.7%)	

Notes: The data are presented using mean ± standard deviation and *n* (%), ^a^ The difference between the PSME group and control group were compared using independent *t*-tests with the level of difference significance set at *p* < 0.05; ^b^ The difference between PSME group and control group were compared using the chi-square test with the level of significant difference set at *p* < 0.05.

**Table 3:** The comparison of outcomes within the 2 groups at baseline, 4 weeks, and 8 weeks.

Variables	Group	Baseline	4 Weeks	8 Weeks
TUGT (s)	PSME	11.9 ± 1.6	10.6 ± 1.4 *	10.4 ± 1.5 *
	Control	11.6 ± 0.8	11.4 ± 1.0	11.1 ± 1.0
OLST: opened eyes (s)	PSME	11.5 ± 10.3	9.3 ± 7.3	11.2 ± 10.1
	Control	16.3 ± 13.7	16.4 ± 12.8	13.3 ± 10.9
OLST: closed eyes (s)	PSME	3.3 ± 3	4.1 ± 2.6	3.9 ± 2.5
	Control	4.8 ± 4.7	4.7 ± 3.3	4 ± 2.8
FTSST (s)	PSME	13.3 ± 3.0	9.4 ± 1.5 *	8.2 ± 1.0 *
	Control	9.2 ± 1.9	9.7 ± 1.8	10.7 ± 2.8 *
2MST (steps)	PSME	62.5 ± 12.3	77.7 ± 11.0 *	84.3 ± 9.6 *
	Control	75.1 ± 15.1	76.5 ± 14.2	80.4 ± 22.4

Note: The data are presented using mean ± SD. Two-way repeated measure ANOVA * = Significant difference between 4 weeks and 8 weeks with baseline set at *p* < 0.05; Abbreviations: TUGT = Timed up and go test, OLST = One leg standing test, FTSST = Five time sit to stand, 2MST = 2-min step test.

**Table 4:** Comparison of the adjusted mean and 95% CI difference of outcome measures (ANCOVA) at each assessment session and effect size between the two groups.

Variables	Group	Baseline	4 Weeks			8 Weeks			Effect Size between Group at 8-Weeks
			Adj. Mean	Difference (95% CI)	*p*-Value	Adj. Mean	Difference (95% CI)	*p*-Value	
TUGT	PSME	11.8	10.5	−1.1 (−1.5 to −0.5)	<0.001 *	10.3	−0.9 (−1.4 to −0.4)	<0.001 *	0.5
	Control		11.5			11.2			
OLST: opened eyes	PSME	8.3	7.9	1.2 (−0.6 to 1.2)	0.363	8.7	1.1 (−1.4 to 1.6)	0.633	0.2
	Control		9.3			7.9			
OLST: closed eyes	PSME	2.8	3.5	1.1 (−1.3 to 1.5)	0.770	3.5	1.2 (−1.2 to 1.6)	0.382	0.1
	Control		3.4			3.0			
FTSST	PSME	10.7	8.4	−1.2 (−1.36 to −1.2)	<0.001 *	7.8	−1.4 (−1.6 to −1.2)	<0.001 *	1.5
	Control		10.4			10.9			
2MST	PSME	68.8	79.8	5.3 (−1.5 to 12.1)	0.127	87.0	9.4 (−0.1 to 18.7)	0.05	0.2
	Control		74.5			77.7			

Note: The data are presented using mean ± SD. Analysis of covariance and effect size at 4 and 8-weeks of exercise * = Significant difference between the two groups at 4 weeks and 8 weeks at a *p*-value < 0.01. Abbreviations: TUGT = Timed up and go test, OLST = One leg standing test, FTSST = Five time sit to stand, 2MST = 2-min step test.

**Table 5:** The comparison of quality of life and fear of falling within the two groups at baseline, 4 weeks, and 8 weeks.

Variables		Group	Baseline	4 Weeks	8 Weeks
Quality of life (scores)	Physical domain	PSME	25.1 ± 3.9	32.0 ± 2.0 *^,†^	30.8 ± 2.7 *^,†^
		Control	27.2 ± 4.7	27.6 ± 5.3	25.8 ± 4.8
	Psychological domain	PSME	20.8 ± 4.8	27.4 ± 2.7 *^,†^	26.2 ± 2.8 *^,†^
		Control	23.8 ± 4.0	24.1 ± 4.4	22.9 ± 4.8
	Social relationships domain	PSME	11.2 ± 3.4	12.9 ± 1.6 *^,†^	11.9 ± 2.2 *
		Control	11.7 ± 2.7	11.1 ± 2.5	10.4 ± 2.7
	Environmental domain	PSME	24.6 ± 15.7	26.7 ± 2.4 *	24.4 ± 3.5 *
		Control	22.3 ± 3.9	22.1 ± 4.0	21.5 ± 3.7
	Overall domain	PSME	92.8 ± 13.6	115.7 ± 8.9 *^,†^	110.4 ± 11.4 *^,†^
		Control	0.3 ± 15.2	00.2 ± 16.7	95.3 ± 17
Fear of falling (scores)		PSME	28.2 ± 5.9	0.2 ± 5.2	21.3 ± 4.3 *
		Control	24.7 ± 8.1	4.9 ± 8.1	26.5 ± 6.6

Note: The data are presented using mean ± SD. Two-way repeated measure ANOVA. * = Significant difference between 4 weeks and 8 weeks with *p*-value < 0.05 in the between group comparison. ^†^ = Significant difference between 4 weeks and 8 weeks with baseline in the within group comparison at a *p*-value < 0.05.

**Table 6:** Comparison of the adjusted mean and 95% CI difference of quality of life and fear of falling (ANCOVA) at each assessment session and effect size between the two groups.

Variables	Group	Baseline	4 Weeks			8 Weeks			Effect Size between Group at 8-Weeks
			Adj. Mean	Difference (95% CI)	*p*-Value	Adj. Mean	Difference (95% CI)	*p*-value	
Physical domain	PSME	26.1	32.4	5.3 (3.3 to 7.2)	<0.001 *	31.3	5.8 (3.8 to 7.7)	<0.001 *	1.3
	Control		27.2			25.4			
Psychological domain	PSME	22.3	27.9	4.4 (2.6 to 6.2)	<0.001 *	26.9	4.6 (2.7 to 6.5)	<0.001 *	0.8
	Control		23.6			22.2			
Social Relationships domain	PSME	11.5	13.0	1.9 (0.9 to 2.9)	<0.001 *	11.9	1.6 (0.3 to 2.9)	0.015 ^†^	0.6
	Control		11.1			10.3			
Environmental domain	PSME	23.4	26.7	4.6 (2.9 to 6.3)	<0.001 *	24.4	2.8 (0.9 to 4.7)	0.004 ^†^	0.8
	Control		22.1			21.6			
Overall domain	PSME	96.5	117.4	18.9 (12.6 to 25.2)	<0.001 *	112.1	18.5 (11.6 to 25.4)	<0.001 *	1.0
	Control		98.5			93.6			
Fear of falling	PSME	26.4	29.1	3.16 (0.4 to 5.9)	0.024^ †^	21.0	−5.7 (−8.7 to −2.8)	<0.001 *	0.9
	Control		25.9			26.8			

Note: Analysis of covariance and effect size at 4 and 8-weeks of exercise. * = Significant difference when compared to baseline with a *p*-value < 0.001. ^†^ = Significant difference when compared to baseline with a *p*-value < 0.05.

## Appendix A

**Table A1:** Progressive step marching exercise choreography.

Movement Pattern	Description
1. Marching in place	Stand straight with hip wide apart and place the arm on the side of the body. Raise right knee with hip and knee flexion approximately 45 degrees then repeat on the opposite side and keep alternating sets. MP total sets is upon music speed which counted by 8 steps as 1 set.
2. Marching forward	Stand straight with hip wide apart and place the arm on the side of the body. Raise right knee with hip and knee flexion approximately 45 degrees. Moving forward for 4 steps then moving backward 4 steps.
3. Marching backward	Stand straight with hip wide apart and place the arm on the side of the body. Raise right knee with hip and knee flexion approximately 45 degrees. Moving forward for 4 steps then moving backward 4 steps.
4. Marching to right side	Stand straight with hip wide apart and place the arm on the side of the body. Raise right knee with hip and knee flexion approximately 45 degrees. Moving to right side of the body for 4 steps then moving backward 4 steps.
5. Marching to left side	Stand straight with hip wide apart and place the arm on the side of the body. Raise right knee with hip and knee flexion approximately 45 degrees. Moving to left side of the body for 4 steps then moving backward 4 steps.
6. Step touch	Stand straight with the arm on the side of the body. Step the right leg to the right side. Move the left leg to the right leg. Keep both legs together. Step the left leg to the left side. Stand straight with the arm on the side of the body.
7. Walking forward	Starting with right leg walking forward then continue by alternating right and left sides (right, left, right and left). Walking backward with alternating right and left sides (left, right, left and right).
8. Walking backward	Starting with right leg walking backward then continue by alternating right and left sides (right, left, right and left) Walking forward with alternating right and left sides (left, right, left and right)
